# Combination of Transcranial Magnetic Stimulation and Ketamine in Treatment-Resistant Depression: A Systematic Review

**DOI:** 10.7759/cureus.64712

**Published:** 2024-07-17

**Authors:** Oluwatosin O Arubuolawe, Ibrahim L Folorunsho, Adeniyi K Busari, Chidalu Ibeneme, Amarachukwu B Diala, Victory I Afolabi, Nkechinyere M Harry, Kenechukwu Anona, Vivien O Obitulata-Ugwu, Olubukola A Kuye, Gibson O Anugwom

**Affiliations:** 1 Psychiatry, Manhattan Psychiatric Center, New York, USA; 2 Psychiatry, General Directorate of Health Affairs, Najran, SAU; 3 Public Health, Emory University, Atlanta, USA; 4 Public Health, University of Toledo, Toledo, USA; 5 Psychiatry, Youngstown State University, Youngstown, USA; 6 College of Medicine, University of Ibadan, Ibadan, NGA; 7 Psychiatry, Vinnytsia National Pirogov Medical University, Vinnytsia, UKR; 8 Psychiatry and Behavioral Sciences, University of Ibadan, Ibadan, NGA; 9 College of Medicine, University of Nigeria, Enugu, NGA; 10 Psychiatry, Obafemi Awolowo College of Health Sciences, Ago Iwoye, NGA; 11 Psychiatry and Behavioral Sciences, Baylor College of Medicine, Houston, USA

**Keywords:** combination tms with ketamine, treatment-resistant depression (trd), major depressive disorder (mdd), depression, ketamine, transcranial magnetic stimulation (tms)

## Abstract

Treatment-resistant depression (TRD) is a significant challenge in psychiatric practice, affecting a substantial proportion of patients with major depressive disorder (MDD). Traditional treatment modalities often fall short, necessitating the exploration of alternative therapies. This literature review examines the combined use of Transcranial Magnetic Stimulation (TMS) and ketamine in treating TRD.

The objective of this study is to evaluate the efficacy, safety, and potential synergies of combining TMS and ketamine in the treatment of TRD.

A comprehensive literature search was conducted using PubMed and Google Scholar databases from 2014 to 2024. The search terms included combinations of "Transcranial Magnetic Stimulation," "Ketamine," "Depression," "Major Depressive Disorder," "Treatment-Resistant Depression," and "Combination." After screening for relevance and applying inclusion and exclusion criteria, six studies were selected for review, including three case reports, a retrospective study, a pilot study, and a review study.

The selected studies demonstrated that the combination of TMS and ketamine resulted in substantial and sustained improvement in depressive symptoms for patients with TRD. Case reports and retrospective studies highlighted significant reductions in depression severity and improvements in psychosocial functioning. The combination therapy showed a higher efficacy compared to monotherapies of either TMS or ketamine alone. Notably, adverse effects were generally mild and transient, with no severe adverse events reported in most studies.

In conclusion, the combination of TMS and ketamine presents a promising treatment modality for patients with TRD, offering significant improvements in depressive symptoms and better outcomes compared to traditional monotherapies. However, the heterogeneity in study designs and small sample sizes underline the need for larger, randomized controlled trials to establish standardized protocols and further validate these findings.

## Introduction and background

Depressive disorder is a chronic condition that causes significant morbidity to many patients [1]. It affects approximately 280 million people globally [1]. There have been several evaluations of the efficacy and failure of antidepressants for major depressive disorders. These studies show that about 15% to 30% of patients with major depression treated with antidepressants will fail to respond to treatment [2].

There is no consensus on the definition of treatment-resistant depression as this may vary widely based on treatment observers. According to the US Food and Drug Administration (FDA) and the European Medicines Agency (EMA), treatment-resistant depression refers to major depressive disorders that fail to respond to two different antidepressants from two distinct pharmacological classes, given at standard doses for at least six weeks despite patient’s compliance with treatment regimen [3,4]. 

Current treatment modalities for TRD include antidepressants only, combining antidepressants, second-generation antipsychotics such as quetiapine or aripiprazole, off-label IV ketamine, intranasal esketamine with antidepressants, electroconvulsive therapy (ECT), repetitive transcranial magnetic stimulation (rTMS), and psychotherapy with standard anti-depressants [3,5]. Each of these treatment modalities possesses distinct advantages, drawbacks, and varying levels of efficacy [3]. However, the Global Health Data Exchange (2023) reports that of the 280 million individuals globally afflicted with depression, as many as 35% are resistant to readily available medications [1]. This high percentage of patients resistant to treatment with antidepressant medications and the side effects associated with these medications underscores the need for more review of alternative treatment methods, such as a combination therapy of ketamine and transcranial magnetic stimulation [6]. 

Combination Transcranial magnetic stimulation with Ketamine (CTK) involves the use of TMS and ketamine to treat major depressive disorder unresponsive to pharmacotherapy and TMS or ketamine alone [2]. These were administered in different ways in reviewed studies and case reports [1]. Ketamine is conventionally utilized as a dissociative anesthetic agent. It is a non-competitive NMDA receptor antagonist which is administered off-label as IV ketamine [1]. Intranasal esketamine combined with a newly initiated anti-depressant was approved by the U.S. FDA and EMA in Europe for treatment-resistant depression in 2019 [6,7]. Both provide rapid relief of symptoms to patients. Depending on the protocol followed, ketamine can be successful in treating depression in up to 70% of patients. Although this approach is novel and produces quick improvements, about 30% of patients remain refractory to treatment [1].

Transcranial magnetic stimulation utilizes brain stimulation to achieve a response for pharmacotherapy-resistant depression [8]. It has about 30 to 90% efficacy in patients diagnosed with TRD [1]. TMS is a non-invasive brain stimulation performed by attaching a stimulation coil to the head which produces an electromagnetic impulse that generates electrical currents which impact the cerebral cortex [8]. TMS delivered in a brief repetitive manner is called rTMS. Low or high-frequency rTMS at a dose of 1 Hz and between 10-20 Hz respectively can be applied to the prefrontal cortex daily between four to six weeks in patients with resistant-treatment depression [8]. The motor threshold (MT) has been utilized to direct the computation of the appropriate TMS dosage for each patient in therapeutic applications. TMS is set at a specific percentage MT, which is a measure of the TMS intensity needed to elicit a peripheral motor response [2].

Interestingly, In 2022, the FDA approved a new protocol called Stanford Accelerated Intelligent Neuromodulation Therapy (SAINT), which utilizes intermittent theta-burst frequencies (iTBS). This protocol involves the delivery of 1,800 iTBS pulses at 90% MT in less than 10 minutes, every hour, 10 times a day for 5 days, resulting in 50 treatment sessions corresponding to 90,000 pulses in 5 days. At the end of treatment, the reported success rate, defined as a reduction of symptoms, was 90%, with a 60% success rate observed a month later [1].

Various studies have examined the use of the combination treatment. Overall, the studies showed a decrease in patients’ depression symptoms and improvement in psychosocial impairment [1,9]. Although no harmful effects were reported in several studies reviewing the combination treatment, it is imperative to exercise caution when using the combination treatment due to the excitatory effect on the prefrontal motor cortex [1]. However, the return of the prefrontal excitability to baseline 24 hours following ketamine administration shown in another study, further shows a reduction in risk of excitability prior to rTMS treatment. 

This study aims to conduct a retrospective literature review focusing on ketamine and transcranial magnetic stimulation combination treatment, specifically exploring their efficacy, safety, adverse effects, and potential synergies in addressing treatment-resistant depression.

## Review

Method

PubMed and Google Scholar were searched for articles between 2014 and 2024 using mesh terms (“Transcranial Magnetic Stimulation" OR "TMS") AND ("Ketamine") AND ("Depression" OR "Major Depressive Disorder" OR "Treatment-Resistant Depression") AND ("Combination" OR "Synergistic"). On PubMed, 13 papers were generated out of which only four articles were relevant to our topic. From Google Scholar, we found 6,540 articles. Among these, nine articles were deemed relevant to our research topic.

No age restriction was applied because of the limited availability of articles related to the topic. All articles were published in English. No specific article type was excluded based on the route of administration of ketamine. Four duplicate articles were excluded from the studies found in both PubMed and Google Scholar. Three articles that do not have full texts were also removed from Google Scholar.

After applying the above inclusion and exclusion criteria, only six papers were relevant and selected for our study. The six articles were independently read by the authors.

Inclusion Criteria

Articles published in English within the last 10 years; clinical studies evaluating the combination of TMS and ketamine; studies involving patients with TRD, MDD and bipolar depression.

Exclusion Criteria

Animal studies or preclinical trials; studies without primary clinical outcomes related to depression treatment; articles without accessible full text.

**Figure 1:**
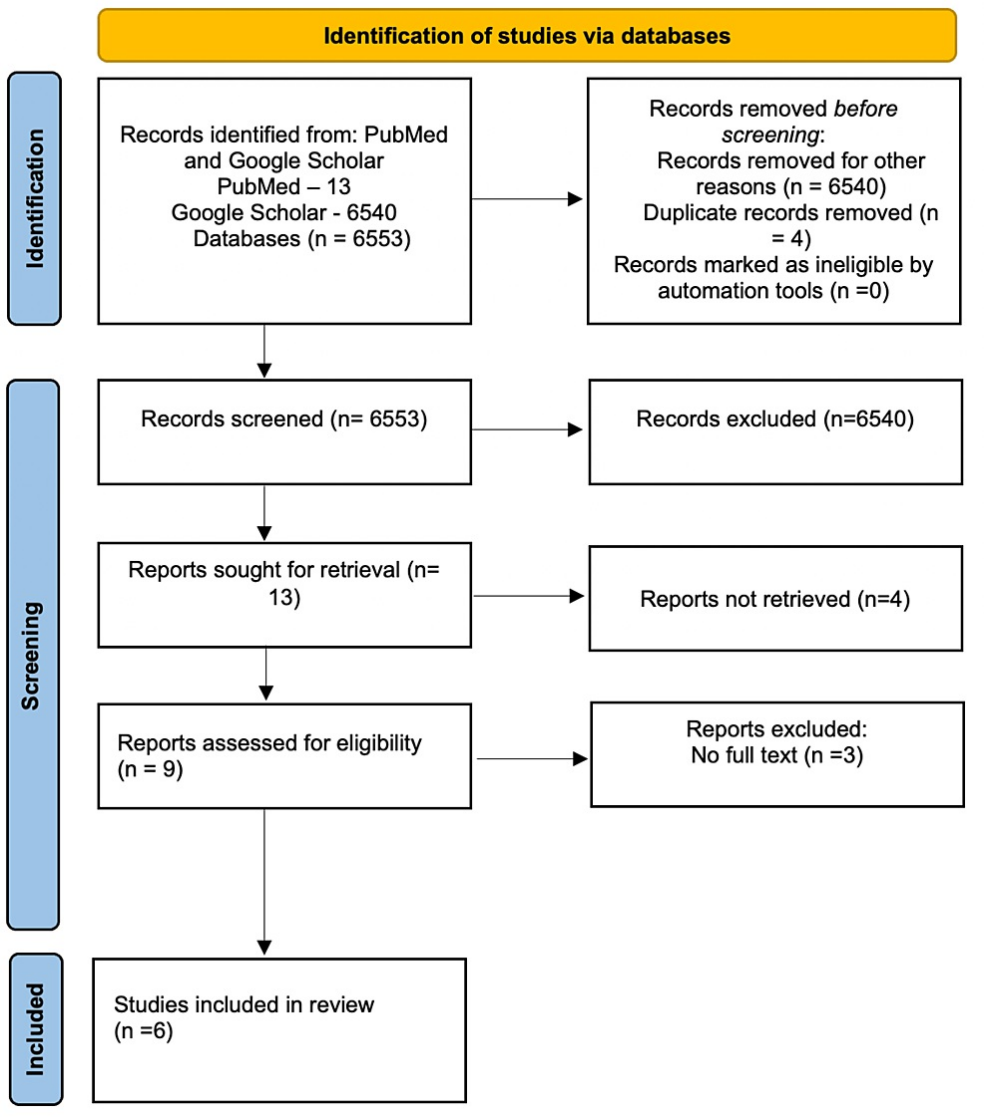
PRISMA diagram encapsulating the process of article search, screening, and inclusions. PRISMA: Preferred Reporting Items for Systematic Reviews and Meta-Analyses.

Figure 1 depicts the process of article search, screening, and inclusions.

Results

Summary of Included Studies

In this review, we found six eligible articles: case reports - three, retrospective study - one, pilot study - one, and systematic review- one that discussed the efficacy, safety profile and the current gap in the literature of combination transcranial magnetic stimulation with ketamine in the treatment of treatment-resistant depression. The summary of the six studies is presented in Table 1.

**Table 1:** Summary of included studies CTK- Combined repetitive transcranial magnetic stimulation (TMS) and ketamine; IV- intravenous; IM- Intramuscular; TRD- treatment-resistant depression; MDD- major depressive disorder; GAD- generalized anxiety disorder; SUD- substance use disorder; OCD- obsessive-compulsive disorder; ADHD- Attention-Deficit Hyperactivity Disorder; CGI-S- Clinical Global Impression-Severity scale.

S/N	Study	Year	Design/study type	Sample Size	Population	Treatment Protocol	Outcomes
1.	Elkrief et al [10]	2022	Case report	1	43-year-old man with bipolar disorder type 1(only one previous manic episode) and TRD. Comorbidity- GAD.	Several sessions of CTK (using rTMS + IV/IM ketamine.) administered for 12 weeks in a stepwise fashion after the monotherapies of rTMS and IV Ketamine failed.	Total and prolonged resolution of depressive symptoms one year after treatment completion.
2.	Stephen Best et al [2]	2019	Retrospective review	28	21-70 years TRD (unipolar and bipolar patients with comorbid, SUD, anxiety disorder and neuropathic pain.	Concurrent administration of TMS and ketamine infusion- 10 to 30+ sessions.	Clinical Improvement in depression symptoms and statistically significant reduction in mean CGI-S score at various points during pre-treatment(T1), post-treatment(T2), and two years after treatment end (T3). (T1→T2: α= 0.01, t=22.81, p<0.0001) and (T2→T3: α= 0.01, t=27.36, p<0.0001).
3.	Stephen Best and Brian Griffin [11]	2014	Case report	1	23-year-old woman with TRD, ADHD, anorexia nervosa and substance abuse (cocaine, alcohol) in full remission.	Combined TMS and Ketamine infusion given after two days of TMS pretreatment. Total of 13 sessions administered weekly.	Substantial improvement in depression symptoms and decreased alcohol use.
4.	Shanok et al [6]	2024	Pilot study	235	TRD patients	169 participants with treatment-resistant MDD received 36 treatment sessions of Deep TMS-only (H1 + H7 protocols), while 66 received 36 treatment sessions of Deep TMS (H1 + H7 protocols) and six IV infusions of ketamine over the course of nine weeks.	Significant reduction in depression symptoms in the TMS only group (P< 0.001) and the TMS + IV Ketamine group (P< 0.001). Comparing TMS only group with TMS + IV Ketamine group, P= 0.13.
5.	Debowska et al [1]	2023	Review study	11 studies	Studies on CTK	PubMed, Web of Science and Google scholar were searched, and relevant articles analyzed.	All CTK studies showed significant improvement in depression symptoms.
6.	Stephen R. D. Best [12]	2015	Case report	1	32-year-old male who has chronic OCD with somatic fixation, depression, and GAD.	CTK administered for 12 weeks.	Significant reduction in depression and anxiety symptoms (including cessation of suicidal ideation) but OCD symptoms remained unchanged.

A case study of a 43-year-old man with severe TRD and a bipolar disorder diagnosis who failed to respond to several trials of TMS and Ketamine monotherapy was published by Elkrief et al in 2022. The baseline Montgomery-Asberg Depression Rating Scale (MADRS) was 38 and Clinical Global Impression (CGI) was five. He agreed to try CTK. Intravenous ketamine (0.5 mg/kg) infusions twice a week and intermittent theta burst stimulation (iTBS) (600 pulses per session/3 sessions per day) administered to the left dorsolateral prefrontal cortex (DLPFC) constituted the first two-week phase, and continuous TBS (cTBS) to the right DLPFC (600 pulses per session in both cases, 48 sessions total) were used during the 10-week consolidation period. Eight intramuscular ketamine doses were also given (once weekly for the first six weeks, then once every two weeks). CTK was not administered concurrently. TMS and Ketamine were spaced four hours apart during treatment. At the end of his treatment, MADRS became four and CGI became one. Six months later, he continued to have sustained remission and he experienced a lot of improvement in his lifestyle. It should be noted that the iTBS procedures employed in this case differ from those used in other studies, in which left-rTMS was the primary method of stimulation [10].

In a retrospective study, 28 patients had a variety of mood disorders with the majority being unipolar depression. They all had something in common - treatment-resistant depression and had tried different treatments prior to this study. Twenty patients received pretreatment with rTMS based on a criterion for phobia behavioral traits. The remaining eight did not receive pretreatment. TMS protocol of 1 Hz 130% MT 30 mins was applied to the medial prefrontal area targeting the ACC. Five minutes after the commencement of TMS, IV ketamine infusions started from 20 mg with an average dosage range of 0.4-2.3 mg/kg over 20 mins until a cataplexy was reached. This is a crucial component of CTK. The number of treatment sessions was based on the responsiveness of the patients. Some had 10-20 while some had more than 30 sessions. The outcome measure was the Clinical Global Impression-Severity (CGI-S) scale. The CGI-S is one of the most commonly utilized brief assessment tools in psychiatry for measuring the effectiveness of treatments for depressive disorders. It provides a relatively objective way to assess remission. The CGI-S uses a seven-point scale to assess the severity of illness, ranging from 1 for normal to 7 for the most severely ill patients. Though, as with all assessment tools, it is not without its limitations. For instance, the CGI scale may be prone to issues with unclear scoring anchors and subjective assessments. This study had two objectives: The first was to find out if CTK resulted in a reduction in CGI-S and the second was to find out if CTK resulted in a sustained reduction of CGI-S values for patients after two years of treatment. Most patients were moderately and severely depressed during pretreatment with a mean CGI- S of 6.1 which reduced to 1.7 post treatment. The average decrease in CGI severity among the patients after CTK was 4.46 ± 0.54 with a 99% confidence level and was considered statistically significant using a paired t-test (α = 0.01, t = 22.81 p < 0.0001).* *The CGI-S was sustained for two years since the mean CGI-S remained 1.7 and further reduced to 1.4 after two years and this improvement was statistically significant based on a second paired t-test (α = 0.01, t = 27.36, p < 0.0001). This shows that the patients improved substantially even two years after treatment [2]. This was the first study to demonstrate sustained improvement after two years of CTK administration. 

Another case report was that of a 23-year-old lady with TRD, comorbid with attention deficit disorder, anorexia nervosa and substance use disorder. She received a pretreatment of rTMS for two days, followed by a combination of rTMS (1 Hz, 115% MT for 40 min at the medial prefrontal cortex to target the anterior cingulate cortex (ACC)) and IV ketamine. The dose of ketamine was gradually increased from 50 mg to 600 mg, 30 min per session for 13 weeks. The treatment led to a significant reduction in depressive symptoms with Beck Depression Inventory (BDI) scales reducing from 17 to zero. Also, improvement was noticed in personality and suicidal scales [11]. The improvement in depressive symptoms was seen up to 483 days post-treatment [11]. This paper is the first to indicate that the combination of ketamine and TMS can be better than individual treatment alone and can also sustain the improvement of depressive symptoms much longer [11].

In another study with 235 participants with treatment-resistant MDD, 169 patients received 36 treatment sessions of Deep TMS-only while 66 patients received 36 treatment sessions of Deep TMS and six IV infusions of ketamine over the course of nine weeks. Depressive symptoms were compared pre-and-post treatment in both groups using the patient health questionnaire (PHQ-9). In both treatment groups, depressive symptoms were significantly reduced from pre-to-post. In the TMS-only group (P< 0.001) and the TMS + IV Ketamine group (P< 0.001). However, there were no statistically significant differences in response rates between the TMS + ketamine group and the TMS-only group (P= 0.13). The TMS + ketamine group had an 80.30% response rate (53 out of 66) and 43.42% remission rate (28 out of 66), compared to a 76.92% response (130 out of 169) and 39.64% remission (67 out of 169) in the TMS-only group. This was the only study that did not show a palpable difference between CTK and monotherapy with either TMS or ketamine alone [6]. 

In a systematic review where 11 articles were studied, there were positive results that showed superior efficacy of CTK over TMS or ketamine monotherapy. Most of the patients had TRD (either bipolar or unipolar). Most papers (nine articles) used the 1 Hz 115% MT 20-40 mins as the TMS protocol combined with ketamine infusion. The majority of the studies combined TMS and ketamine infusion concurrently while only two cases did not administer TMS and ketamine simultaneously. One of the non-concurrent cases involving a patient with bipolar disorder had six ketamine injections over two weeks, preceded with rTMS with a protocol of 10 Hz 120% applied to the left DLPFC which does not follow the principle of combination TMS with ketamine model that has been described. This case had only a 19% response rate and 9.5% remission. This is not as significant as others. Another non-concurrent case involved a patient with unipolar depression, who had an initial two weeks of three iTBS per day, twice a week and a consolidation phase of 48 sessions (cTBS for 10 weeks and iTBS, three-daily session twice a week for six weeks) with eight intramuscular ketamine injections (once a week for six weeks, then every two weeks). This case demonstrated a complete and sustained remission of depression symptoms [1].

In another case report, patient X, a 32-year-old man, presented with somatic fixation, depression, and generalized anxiety disorder in addition to persistent obsessive-compulsive disorder. Prior to commencing ketamine/TMS treatment, Patient X underwent 50 months of psychotherapy, antidepressants and antipsychotics. The Beck Hopelessness Scale (BHS), the Beck Anxiety Inventory (BAI), the Beck Depression Inventory-II (BDI-II), and the Personality Assessment Inventory (PAI), which has subscales evaluating several psychopathology categories, were all included in the evaluation. Scores on the PAI subscale higher than 70 imply issues that are clinically significant. Patient X showed severe depression (BDI-II = 48) at the pretreatment assessment in terms of cognitions (PAI DEP-C = 81), affect (PAI DEP-A = 77), and hopelessness assessments (BHS = 18). Furthermore, patient X reported experiencing extreme anxiety (BAI = 52). Patient X received two days of rTMS pretreatment prior to starting combination treatment. The next day, CTK treatment was started and was administered twice a week for 10 weeks. Intravenous ketamine infusion was given concurrently with and bracketed within the middle 40 minutes of TMS, resulting in five minutes of TMS pre and post infusion. The combined therapy consisted of 50 minutes of 1 Hz continuous TMS 115% of MT. Patient X underwent a thorough evaluation in the post-treatment phase, conducted by the same professional psychologist. Patient X reported significantly lower levels of depression (BDI-II = 13) at this evaluation. There were improvements in cognitions (PAI DEP-C = 61), affect (PAI DEP-A = 66), and assessments of hopelessness (BHS = 4). Furthermore, Patient X reported a decrease in anxiety (BAI = 24). However, the obsessive-compulsive symptoms of Patient X were persistently severe (PAI ARD-O = 84) [12]. This case also shows a reduction in depressive symptoms.

Discussion

The use of combination repetitive transcranial magnetic stimulation with ketamine (IV/IM) led to a substantial and sustained improvement of symptoms in all patients with treatment-resistant depression in all six articles in this review. Only one of the studies showed no significant difference between the CTK and the rTMS-only groups [6]. This is understandable because TMS monotherapy has a high efficacy of 30%-90%. From the conclusion of the studies we reviewed that had a significant reduction in depressive symptoms, it was noted that the patients involved in the CTK therapy were refractory to all available treatments for TRD. This could be an important criterion to consider when offering CTK to patients.

The studies show that CTK is very beneficial for persons who have failed or inadequate response to monotherapy of either rTMS or ketamine. Also, it is important to note that CTK resulted in a long-term remission of depressive symptoms for one to two years [2,10]. In Elkrief et al. study, it was noticed that bipolar disorder is somewhat resistant to monotherapy [10]. We have also observed that depression with background comorbidities shows a significant response to CTK and sustained remission for at least a year. For example, a case report by Stephen Best showed that CTK improved symptoms of TRD and anxiety disorder. However, OCD symptoms remained persistent [12].

Adverse effects reported during CTK therapy included nausea, vertigo, local discomfort, which were brief and occasional, and the inconvenience of fasting. The majority of the patients had short-lived psychedelic experiences that were benign. The intensity of psychedelic experiences was dose-dependent and often involved visual distortions. Individuals reported more fascinating internal imagery as the dose increased, such as vivid dream-like experiences. At the highest doses, patients not only endured the psychedelic experience but also found them helpful in overcoming psychological barriers and breaking free from repetitive patterns [2]. Also, during the treatment phase of another study, mood lability as a result of relationship challenges was reported [12]. Further review demonstrated that there was no adverse effect emanating from a combined treatment with TMS plus ketamine in two of the six studies [1,10]. Likewise, in another two of the six studies, it was not mentioned whether combined treatment had any adverse effect [6,12]. Interestingly, there was no patient drop-out during CTK therapy in all the studies reviewed.

Regarding the adverse effects of CTK compared to monotherapy, one case report documented occasional intense dissociation and some nausea during ketamine infusion and none was noticed for the combined treatment [10]. In the same vein, a review study reported lightheadedness/dizziness, sleep irregularities and mania or hypomania as adverse effects of monotherapy with either rTMS or ketamine alone but also did not find any side effect of combined treatment with TMS and ketamine [1]. In three studies representing 50% however, nothing was mentioned regarding the adverse effects of monotherapy [6,11,12].

The cause of depressive disorders is not fully understood. However, there is a common ground that describes the disordered pathways of the cortical, subcortical, and limbic systems. Neurotransmitters and molecular mediators are also involved [13]. Particularly, prefrontal abnormalities on the left side of the brain are associated with unipolar depression. Reduced neuronal activities are seen in the dorsolateral prefrontal cortex (PFC) regions and in the rostral anterior cingulate cortex (ACC) areas which are closely connected together in the brain [14].

TMS mostly targets the dorsolateral prefrontal cortex (DLPFC) [15]. There are different treatment protocols for TMS. One is repetitive TMS (rTMS), and the other is intermittent TMS (iTMS/Theta burst stimulation/TBS). Different studies have shown that high-frequency (10 Hz) rTMS applied to the left DLPFC and low-frequency (1 Hz) rTMS delivered to the right DLPFC are effective in reducing clinical depression in depressive populations [16]. Low-frequency rTMS (1<Hz) inhibits the regional cortical activity thereby suppressing motor, sensory or cognitive function. Conversely, high-frequency rTMS (>1 Hz) does the opposite by activating cortical regional activity [17]. The majority of rTMS studies in major depression apply high-frequency stimulation on the left DLPFC [18]. The United States Food and Drug Administration (FDA) approved the application of rTMS to the prefrontal cortex over four to six weeks (20-30 sessions) for the management of treatment-resistant depression [2]. There is experimental support for a connection between MDD and ACC. When rTMS targets ACC stimulation, patients' depression significantly improves compared to sham stimulation or butterfly rTMS [2]. The medial prefrontal region that covers the ACC is the target of the TMS head-coil in CTK, which is positioned on the patient's head and directed mid-sagittal [2]. rTMS generates electric currents in the neuronal circuits. These lead to neural changes such as neurogenesis and synaptogenesis in the hippocampus. Also, synaptic plasticity is seen in the prefrontal cortex and glutamate release in both areas [19]. It has also been shown that high-frequency rTMS can increase brain-derived neurotrophic factor (BDNF) production in the areas of prefrontal cortex and hippocampus [20].

The mechanism of action for ketamine is notably its inhibition of the N-methyl-D-aspartate receptor (NMDAR) on GABAergic interneurons which leads to the production of glutamatergic neurotransmission. This is how it creates its antidepressant effects [21]. Ketamine, just like TMS, has also been demonstrated to produce neural plasticity in cortical and subcortical structures through the upregulation of neurotrophic factors such as BDNF and mTOR (mammalian target of rapamycin) [22]. Ketamine's usual dose used in the treatment of TRD is 0.5 mg/kg per 40 mins, which translates to a peak plasma level of 70-200 ng/ml of ketamine concentration. This is a subanesthetic level. General anesthetic effects are in the range of 2000-3000 ng/ml of ketamine concentration [23]. Ketamine has not been approved by the FDA for depression although it is used as an off-label medication.

TMS and ketamine have different mechanisms of action and target the brain differently but may still act in a complementary manner. These two treatments promote neuroplasticity and synaptic activity and induce neural changes in neurotransmission. Neuroplasticity is the brain’s ability to reorganize and generate new neuronal connections which brings about recovery in mental health disorders [1]. Their synergistic role potentiates the effect of each treatment leading to a more profound and lasting therapeutic effect [1]. Both treatments synergistically potentiate the production of BDNF which is key to neuroplasticity. The increased production of glutamatergic neurotransmission in the brain also contributes to the increased effectiveness of the combined treatment [1].

Compared to the higher frequencies (10-20 Hz) usually used for clinical treatment of depression when TMS is administered alone, CTK delivers TMS at a comparatively lower frequency (1 Hz) but higher intensity [2]. Low frequency TMS usually has an inhibitory effect which results in reduced excitability of the cortex. TMS administration in CTK is applied to the medial prefrontal region that covers the ACC which is the target [2]. Using high values above 130% MT or high-intensity MT allows for the application of the highest coil output a person could tolerate without experiencing severe discomfort. This is because the TMS stimulation point was judged to be far enough away from the motor cortex [2]. Also, the analgesic effect of ketamine in CTK dissolves any painful effect and makes it possible to apply TMS of unusually high intensity [2]. This is probably why CTK is more tolerable and has less adverse effects from the studies above.

Limitations

The heterogeneity in the study designs and population of this literature review is high. This is expected because of this study type. Therefore, a meta-analysis is not feasible at this time, due to the paucity of research on this topic and the wide range of methodologies used in the studies that exist. Additionally, the publications that are currently accessible have limited sample sizes.

Also, CTK is not approved by the FDA, so there are no standard treatment protocols/parameters currently. For example, the stimulation site for rTMS, stimulation frequency, the duration and number of sessions, concurrent/nonconcurrent administration of TMS and ketamine, route of administration of ketamine, dual Ketamine infusion and dual deep TMS protocol. This will make it difficult for researchers to determine the most effective protocol.

In the study by Shanok et al., numerous participants were concerned regarding the use of psychedelic anesthetic medication- ketamine [6]. This led to uneven sample sizes in the two treatment groups, which could affect the study results. With the dissemination and presentation of additional studies on the safety and effectiveness of ketamine treatments in a variety of media, perhaps the public's perspective of the drug will shift toward one that is more positive. 

Moreover, during this review, we discovered a lack of unified and robust outcome measures, such as using different depression-rating scales, e.g., MADRS, CGI, PHQ-9, etc.

Lastly, the neurobiological basis of CTK is not yet fully understood. Randomized controlled trials will be needed to confirm the efficacy and sustainability of this novel treatment, validated by comparing it with the effectiveness of rTMS or high-dose ketamine infusions alone. Large RCTs will also allow for standardizing the protocols for maximum efficacy and safety.

## Conclusions

From the studies above, we infer that combination TMS with Ketamine (CTK) is very efficacious in patients who had failed/inadequate response to monotherapy of either TMS or ketamine alone. CTK should be offered to patients who have unipolar or bipolar major depression who fail to respond to monotherapy. TRD has had a significant response and sustained remission to CTK for at least a year. We have also seen that depression with other comorbidities responds to CTK. Furthermore, combination TMS with ketamine has been found to have a better and more tolerable adverse effect profile compared to monotherapy with either TMS or ketamine alone. Some studies report no adverse effects at all while others mentioned mild side effects ranging from brief nausea and mild but brief vertigo, etc. It is quite intuitive that the reassuring side effect profile of combination TMS with ketamine makes patients’ compliance to therapy easy as there was no patient dropout in all the studies reviewed in this article.

Despite the efficacy, safety profile and the promising nature of CTK, more research and clinical trials with a large sample size and robust outcome measures need to be done to extensively study the efficacy and adverse effects of this combination therapy and develop specific protocols and guidelines for administering CTK rooted in solid scientific evidence.
